# Transcutaneous Radiofrequency Plus Therapeutic Exercise for the Treatment of Diastasis Recti Abdominis After a Twin Pregnancy: A Case Report

**DOI:** 10.7759/cureus.83362

**Published:** 2025-05-02

**Authors:** Paula Cordova-Alegre, Jorge Alamillo-Salas, Carolina Jiménez-Sánchez, Daniel Sanjuán-Sánchez, Beatriz Carpallo-Porcar

**Affiliations:** 1 Faculty of Health Sciences, Universidad San Jorge, Villanueva de Gállego, ESP; 2 Department of Physical Medicine and Rehabilitation, Hospital Royo Villanova, Zaragoza, ESP; 3 Faculty of Nursing and Physiotherapy, Universidad de Lleida, Lleida, ESP

**Keywords:** diastases muscles, diastasis recti abdominis, hypopressive exercises, therapeutic exercise, transcutaneous radiofrequency

## Abstract

Diastasis recti abdominis (DRA) is a common postpartum condition characterized by separation of the rectus abdominis muscles along the linea alba (LA). This condition can lead to significant physical and functional impairment. Conventional physiotherapy interventions, such as targeted abdominal exercises, have been reported to be effective in improving abdominal function. However, the transcutaneous radiofrequency diathermy (TRD) in the treatment of DRA has not yet been explored in this pathology.

This case study reports on the treatment of a 29-year-old woman with a severe supraumbilical DRA (6.5 cm at rest) following a twin pregnancy. The intervention combined conventional physiotherapy and TRD over 13 weeks. The therapeutic exercise program included hypopressive exercises and targeted abdominal muscle strengthening, while the TRD aimed to enhance tissue regeneration and reduce the intra-rectus distance (IRD). The outcomes were the IRD, the distortion index (DI), the involuntary pre-activation of transversus abdominis, and the Physical Function Scale (PF-10).

A significant reduction in the IRD was observed after eight weeks of physiotherapy, at rest (-38%), supraumbilical contraction (-33%) and infraumbilical contraction (-38%). At the end of the TRD intervention, the IRD at rest decreased from 6.5 cm to 2.1 cm (-47.5%), supraumbilical contraction (-64%) and infraumbilical contraction (-64.25%). The distortion index of the LA improved by 50%. Additionally, the patient's physical function, measured using the PF-10 scale, clinically improved.

This case study suggests that conservative physiotherapy based on therapeutic exercises and TRD may be an effective treatment for DRA, especially in severe cases following twin pregnancies. However, larger studies are needed to confirm these findings and to establish TRD as a standard adjunctive therapy in the management of DRA.

## Introduction

The linea alba (LA) is an aponeurotic tissue that is located in the midline of the abdomen and runs from the xiphoid to the pubis. It inserts the deep and superficial abdominal musculature, and its main function is to stabilize the abdominal girdle and contribute to the movement of the trunk [1]. Diastasis recti abdominis (DRA) is abnormal separation of the rectus abdominis muscles along the LA due to increased intra-abdominal tension. The diagnosis can be made by ultrasound, calipers, and palpation, with ultrasound having the best reliability [2]. Regarding the cut-off point for the diagnosis of DRA, Candido et al. [3] categorized DRA as mild if the differential intra-rectus distance (IRD) was greater than 2.5 cm during a curl-up. Mota et al. [4] reported that the range of normal values in women at six months postpartum was between 17 mm and 28 mm, with higher values in multiparous women. It varies among studies because there is no consensus on the IRD cut-off point for diagnosis and depends on the diagnostic method and the sites used for measurement (single or multiple, above, in, or below the umbilicus).

The reported prevalence of DRA is 60% in six weeks and 32.5% at 12 months postpartum, the separation of the rectus abdominis in the supra-umbilical region being more predominant. Pregnancy, maternal age, body mass index, hormonal factors, and twin pregnancy are the main risk factors [5]. Due to DRA, there is a decrease in the integrity, mechanical control, and functional strength of the abdominal wall, which modifies the biomechanics of the trunk, causing pelvic instability and changes in posture, which in turn lead to persistent lumbar-pelvic pain in up to 38% of patients [6]. It is estimated that four out of ten women experience lumbo-pelvic pain at six months postpartum [7]. Moreover, it is also a risk factor for the development of hernias in the midline due to the tissue damage that occurs in LA.

Therapeutic exercise has shown great benefits in DRA reduction, which is the exercise of the transversus abdominis (TrA), and pelvic floor muscles are the most recommended for women with postpartum [8]. The systematic review by Gluppe et al. shows how abdominal exercise reduces DRA [9]. 

In physiotherapy, transcutaneous radiofrequency diathermy (TRD) is a non-invasive technique consisting of the emission of high-frequency electromagnetic waves, which is booming to treat musculoskeletal disorders. TRD leads to an increase in temperature deep within the tissue, improving blood flow or oxygen consumption and speeding up cell activity [10]. Thus, this technique offers myorelaxant, tissue regeneration, and wound healing benefits. Current studies hypothesize that the use of TRD produces a synergistic reduction in the thickness of abdominal fat and an increase in the thickness of the abdomen [11].

There are several studies on DRA in nulliparous women, but from a physiotherapy perspective, there are hardly any studies looking at DRA in twin pregnancies with patients without other risk factors. Secondly, there is an initial diastasis of 6.5 cm at rest, which is rare. Furthermore, to our knowledge, there is no study about the efficacy of TRD plus therapeutic exercises in DRA.

## Case presentation

A 29-year-old woman with a supraumbilical IRD of 6.5 cm at rest after a twin pregnancy was sent for physiotherapy. She is a nurse with no relevant medical history, except for autoimmune hypothyroidism. Prior to the start of the treatment, the patient signed an informed consent form, and the consent of the two collaborating physiotherapy clinics (SPZ clinic and EC clinic) where the intervention was performed was obtained. The treatment lasted 13 weeks (8 in SPZ; 5 in EC). This case report followed the recommendations of the CARE guideline.

The patient gave birth by emergency caesarean section. She gave birth to two sons. The weight of the first child was 2.752 kg and that of the second child was 2.680 kg. The patient had a pre-pregnancy weight of 52 kg and was 163 cm tall. The pregnancy weight was 58 kg and 53 kg after the birth.

Diagnostic assessment

**Figure 1:**
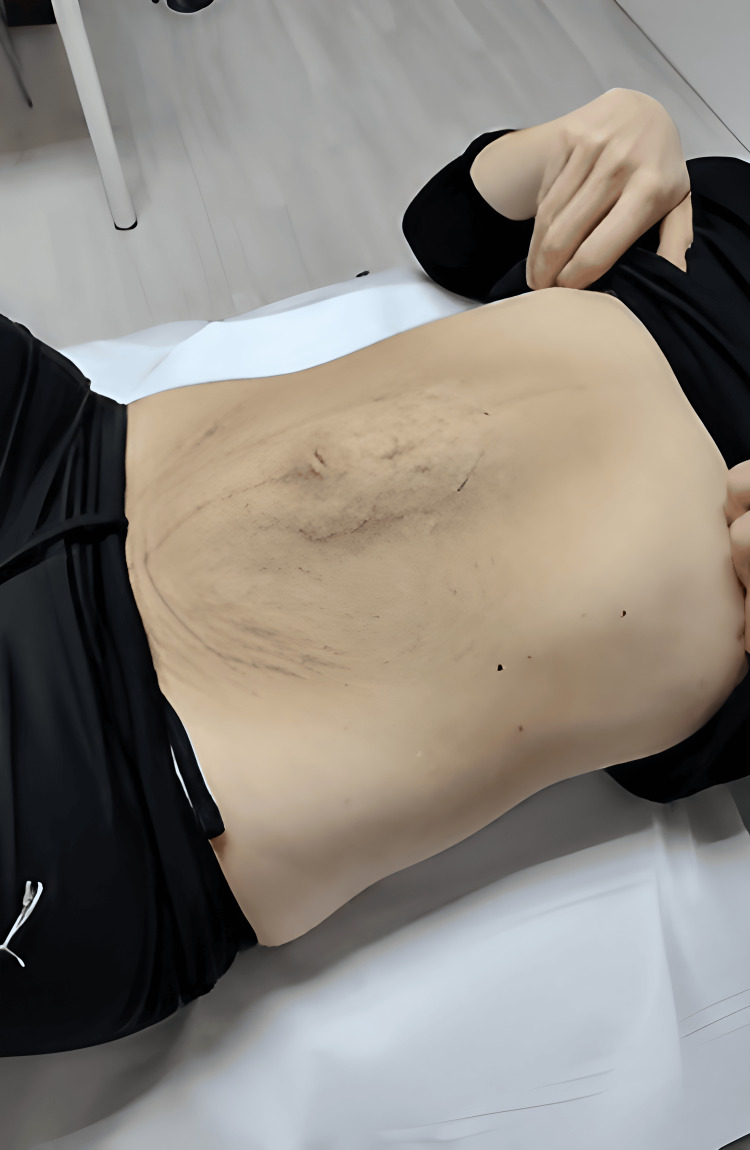
DRA with a minimal abdominal contraction. DRA: Diastasis recti abdominis

The initial DRA diagnosis was performed manually at the SPZ clinic and measured with a caliper. This DRA ran from the xiphoid to the pubic bone. An abdominal bilge was observed in the midline of the anterior abdomen in a light neck flexion (Figure 1).

Physiotherapy management

The patient was treated for eight weeks with one session per week, which the patient supplemented with two sessions of therapeutic exercises at home. The intervention consisted of one-hour sessions, with 30 minutes of hypopressive exercises with rib opening and 30 minutes of abdominal control exercises, strength of both the TrA and the entire abdominal-lumbosacral-pelvic girdle according to current recommendations [12].

The first four sessions consisted of hypopressive exercises in the supine position and quadruped exercises with ribcage opening and abdominal muscle control exercises in supine, quadruped and lateral decubitus were performed. The aim was to incorporate several movements of the lower and upper limbs while maintaining activation of the TrA. Therefore, they were always performed under the supervision of a physiotherapist. In this phase, a neuromuscular bandage with kinesiotape was applied to improve proprioceptive control of the abdominal girdle, which could contribute to a reduction in the DRA. She also received treatment to voluntarily reactivate the TrA. In February, the last four sessions of conventional physiotherapy consisted of 15 minutes of hypopressive exercise in the standing position without rib squeezing, 15 minutes of core exercises according to the 5P® method and 30 minutes of abdominal and TrA strength exercises coordinated with breathing using the "Winner Flow" device. The 5P® method is based on activating the postural tonic system by postural adjustment on an unstable surface [13].

The TRD treatment started one month later and comprised a total of 10 sessions (five weeks) performed by a physiotherapist. Each session lasted 20 minutes and took place twice a week using the "EasyTech T Care Power device". In each session, the base plate was placed on the dorsal-lumbar area and the active heads were moved across the abdomen. The frequency used was 500 Hz in continuous mode with a resistance of 60-80 ohms. The intensity was at the level of hyperthermia (4/5). Each session consisted of a five-minute capacitive TRD phase followed by a 10-minute resistive TRD phase to stimulate the formation of new collagen bridges along the entire LA. In this resistive phase, the patient performed hypopressive exercises without rib opening, with activation of the transversus abdominis muscle and trunk flexion isometrics to increase mechanical stimulation during treatment. The last phase consisted of five minutes of capacitive TRD to stimulate lymphatic drainage. This treatment was complemented by three days/week of the recommended therapeutic exercises at home.

Outcome measures

The first assessment (T0) took place on day 1, and the second assessment (T1) was performed on the first day of TRD on 27 February. The next assessment (T2) was performed in the third week and the last assessment on the last day of TRD treatment (T3).

The IRD was measured manually with calipers on day 1 (T0) at rest at 2.5 cm supra-imbilical and during contraction at two points, at 2.5 cm supra-umbilical and 2.5 cm infra-umbilical. The contraction consisted of an abdominal crunch with a head lift. The patient was in supine position with hips and knees in 90º of flexion, feet resting on the stretcher and arms alongside the body. Ultrasound measurement is the most reliable and valid tool for the diagnosis of this pathology [2]. The ultrasound scanner G.E. LOGIC V2 with a 5-15 MHz 12L-RS linear transducer was used. The transducer was placed transversely at the same points as in the first measurement and in the same position at the end of exhalation [14].

The degree of distortion (DI) of the LA was assessed at T1, T2, and T3. For this measurement, the images of the IRD were acquired in contraction. The distortion area (DA) was calculated with the ImageJ software according to the formula of Lee and Hodges [12] (DI= LA distortion area/shortest distance between lines). The smaller the index, the smaller the LA deformation.

Involuntary pre-activation of the transversus abdominis muscle was assessed at T1, T2, and T3. During a small abdominal crunch, the preactivation was measured as a dichotomous variable (yes/no) with the same transducer device placed 2 cm internal to the ESIA with the linear probe parallel between the ESIA and the iliac crest.

Physical function related to quality of life was measured using the PF-10 scale at T0 and T3. This is a validated and reliable subscale of the SF-36 scale consisting of 10 self-reported items that assess the degree of limitation (1 = very limited, 2 = somewhat limited; 3 = not at all limited) of physical functioning, with a higher score indicating greater physical function. The items are ordered from the most difficult to the easiest. The maximum value of 30 points would be reached if the patient has no limitations in the tasks. The lower value of 10 points would apply when the patient is very limited in the tasks [15].

Results

**Figure 2:**
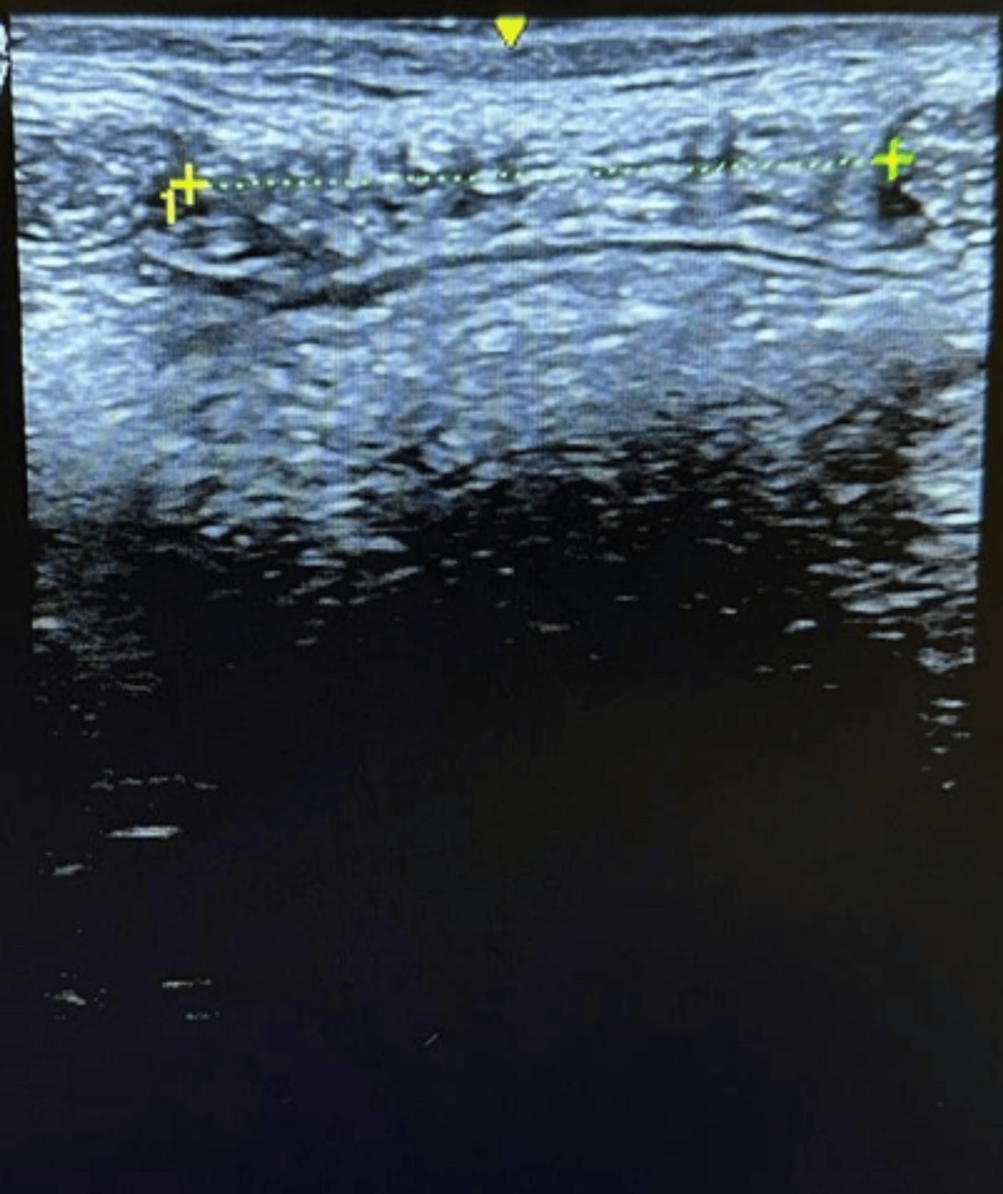
IRD at rest T1 infraumbilical 2.77 cm. IRD: Intra-rectus distance; T1: Post-conventional physiotherapy treatment

**Figure 3:**
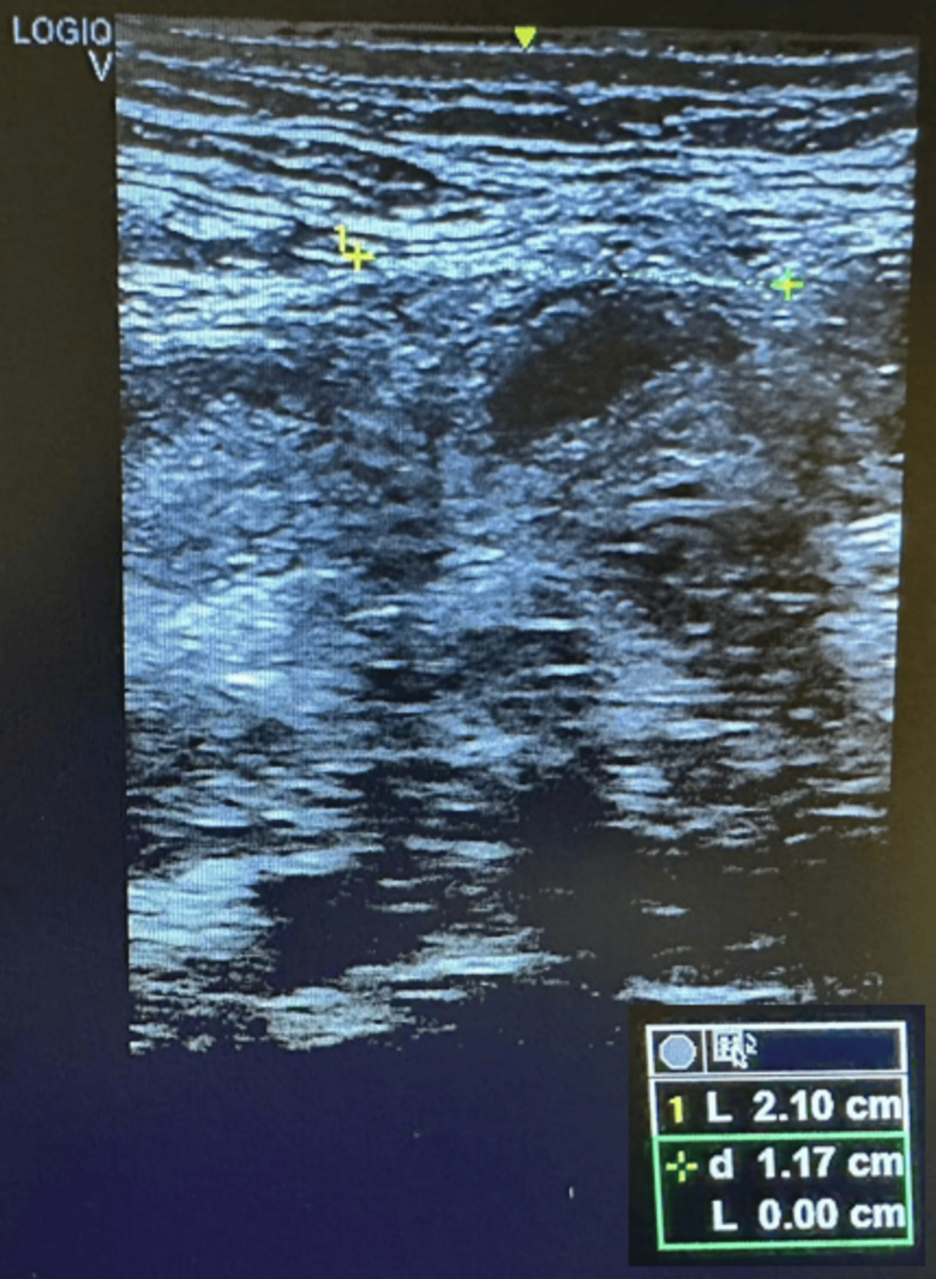
IRD at rest T3. IRD: Intra-rectus distance; T3: Final assessment

**Figure 4:**
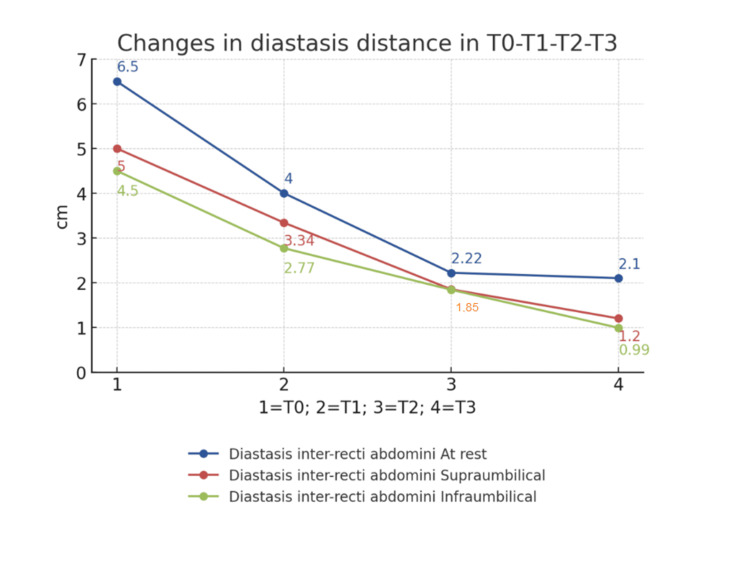
Changes in the IRD. IRD: Intra-rectus distance; T0: Pre-intervention assessment; T1: Post-conventional physiotherapy treatment; T2: Three weeks after transcutaneous radiofrequency diathermy intervention; T3: Final assessment.

**Figure 5:**
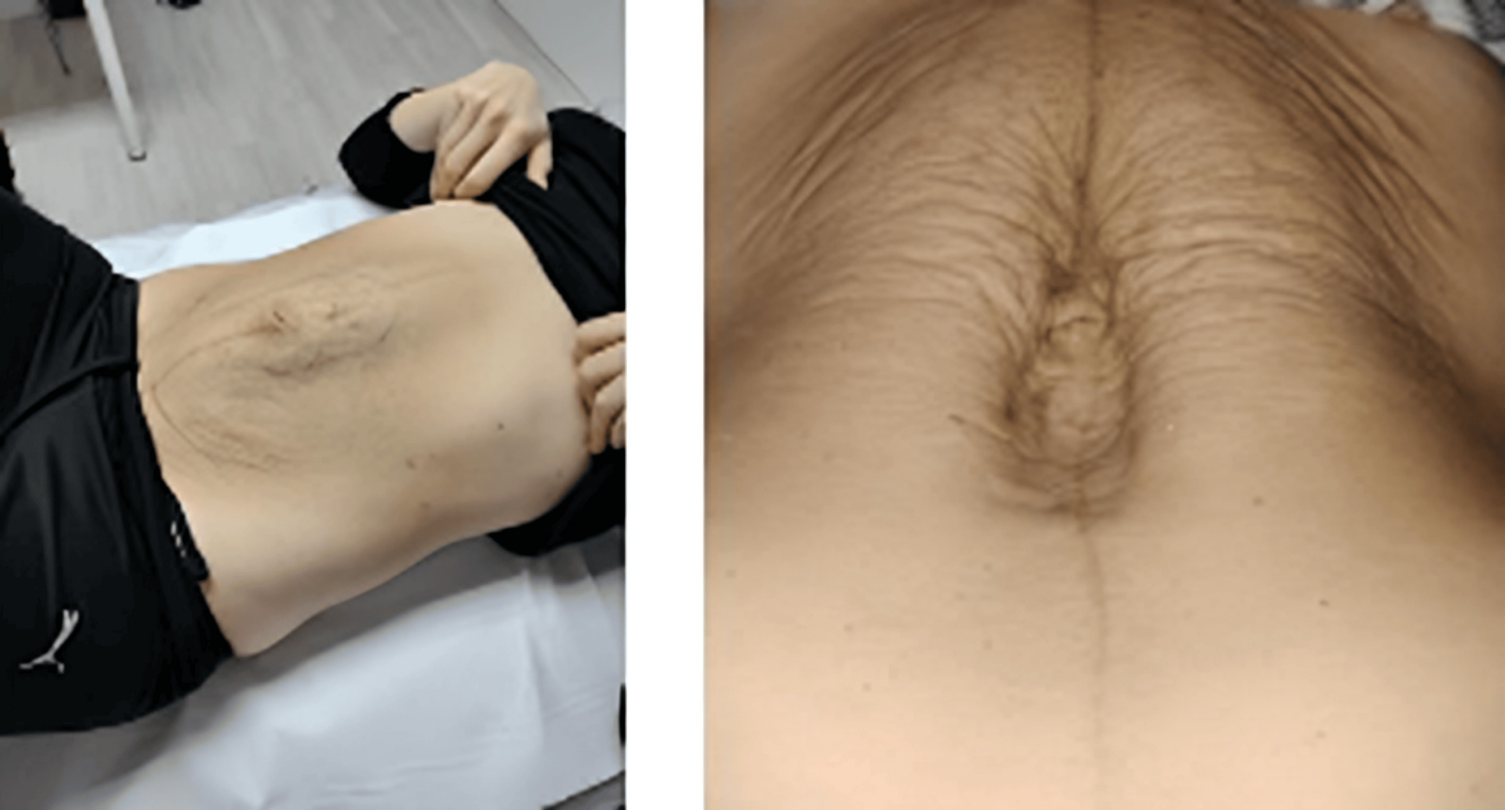
Changes in the DRA function, before, with a large separation and bulge, and at the end, narrower and without bulge. DRA: Diastasis recti abdominis

At the first measurement (T0) of the IRD, the supraumbilical resting distance was 6.5 cm. In the abdominal crunch, the supraumbilical distance was 5 cm and the infraumbilical distance was 4.5 cm. After eight weeks of physiotherapeutic treatment (T1), the distance was 4 cm at rest (-2.5 cm; -38%), 3.34 cm supraumbilically (-1.66 cm; -33%) and 2.77 cm (-1.77 cm; -38%) infraumbilically (see Figure 2). In T2, the supraumbilical distance was 2.22cm at rest (-1.78 cm; -45%), in the abdominal crunch 1.85 cm supraumbilically (-1.49; 45%) and 1.84 cm infraumbilically (-0.93;51%). After the last TRD session (T3), the distance at rest was 2.1 cm (see Figure 3) (-0.12; -5%) and at active 1.2 cm (-0.55; 35%) and 0.99 cm (0.85; -46%) supra- and infra-umbilical, respectively. From T1 to T3, a reduction in rest of 1.9 cm (-47.5%) and at active, 2.14 cm (-64%) supraumbilical, and 1.78 cm (-64.25%) infraumbilical was noted. These data show that the diastasis became functional at activation (Figures 4, 5).

**Figure 6:**
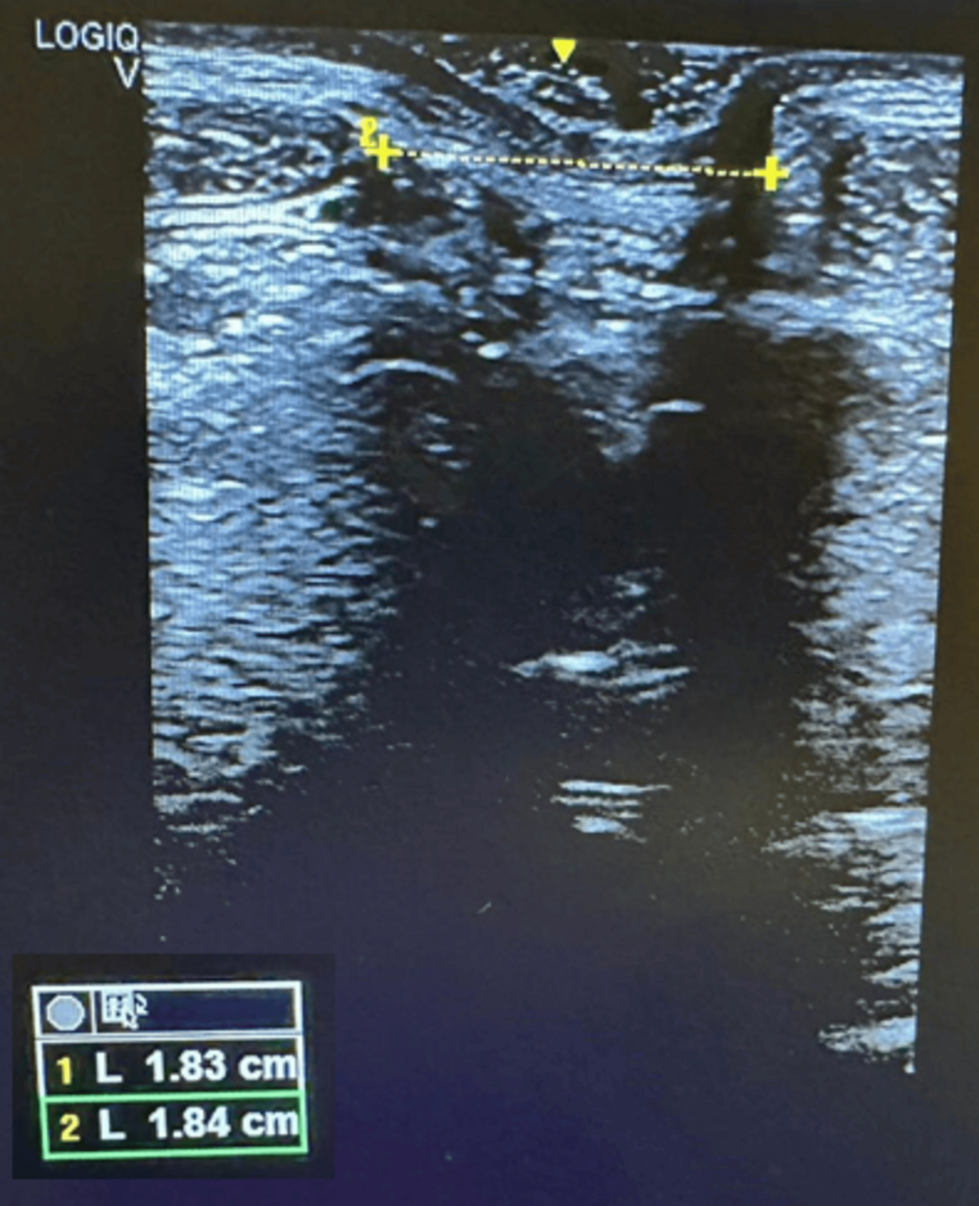
Changes in the supraumbilical linea alba distortion at T1. The arrows indicate the inter-recti distance (IRD), which is the separation between the two rectus abdominis muscles.

**Figure 7:**
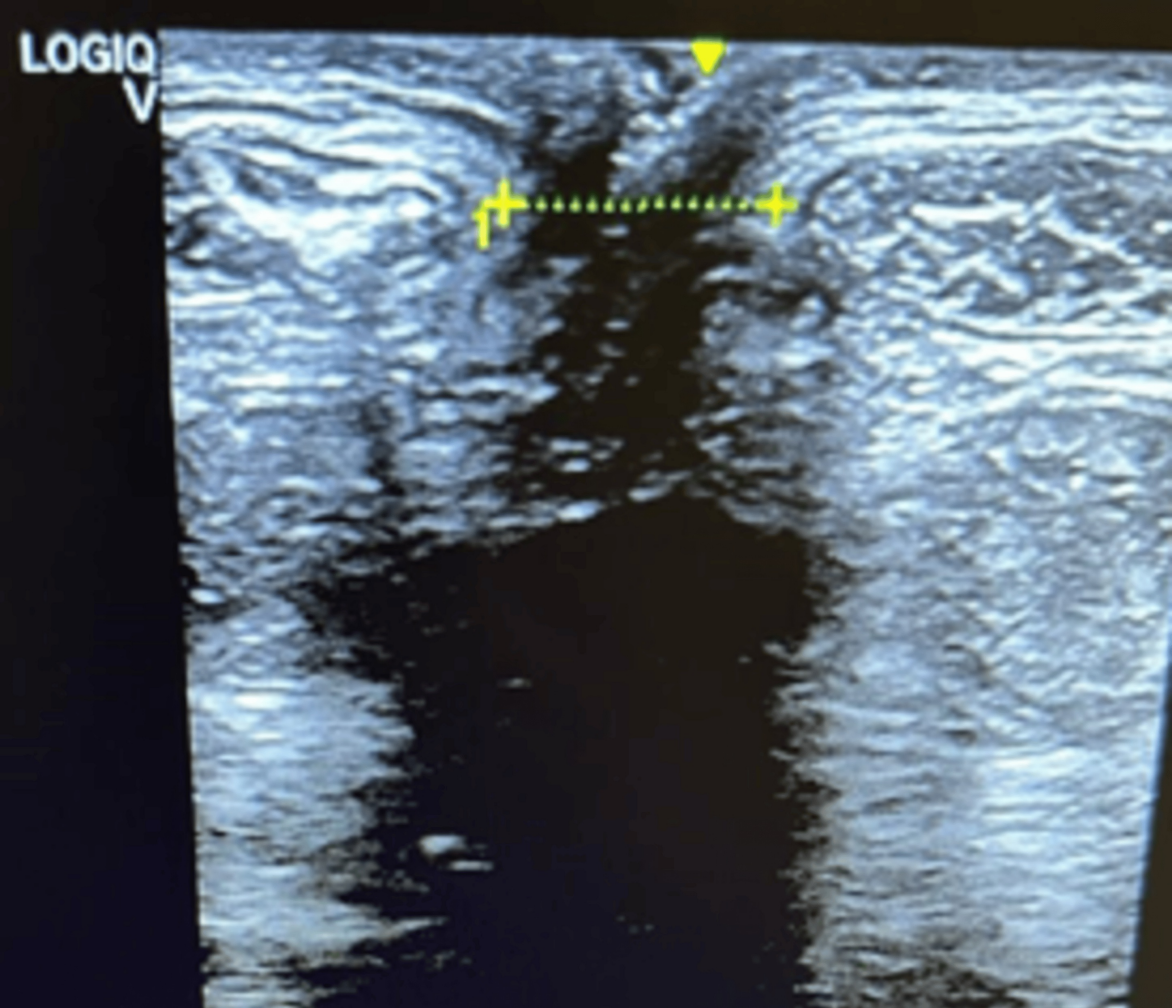
Changes in the supraumbilical linea alba distortion at T3 (0.99 cm) The arrows indicate the inter-recti distance (IRD): separation between the two rectus abdominis muscles.

The supraumbilical distortion index (DI) at the first measurement (T1) was 69.29% (Figure 6). At the second measurement (T2), the distortion was 27.15% and at the last session (T3) 18.48%, which shows an improvement in the quality of the LA after the TRD intervention (Figure 7).

With regard to the pre-activation of the transversus abdominis muscle, an anticipation of the transversus abdominis was observed from the second assessment (T2), which was maintained in the third evaluation (T3).

At the initial assessment (T0) of physical function related to quality of life on the PF-10 scale, the patient had a score of 21 out of 30 points. At the final assessment (T3) after 13 weeks of the program, the patient had a score of 27 out of 30 points. The patient's quality of life improved from six points on the scale.

## Discussion

This case study is the first to demonstrate the potential efficacy of therapeutic exercises combined with TRD in reducing the IRD, DI, and quality of life. Therefore, this case could be a starting point for further research in this area.

After eight weeks of conventional rehabilitation, a reduction in the IRD of 2.5 cm at rest (38%), 1.66 cm during supraumbilical contraction (33%), and 1.77 cm during infraumbilical contraction (38%) was observed. The systematic review by Weingerl et al. [12] showed that abdominal exercise programs are generally effective in the treatment of DRA. In addition, our patient underwent isometric core exercises and progressive rectus abdominis contraction. This treatment has been shown to be effective in this case, as in the study by Simpson et al. [16], in which after 6 and 12 weeks, the exercise that included rectus abdominis contraction achieved greater closure of the DRA than the group that included only TrA contraction. Similarly, in the study by Thabet et al. [8] in which the experimental group performed deep core exercises, the IRD improved significantly compared to the traditional abdominal muscle group. In the same randomized trial, this group also showed significant improvements in the physical function variable measured by the PF-10. Although there is no consensus in the literature on the long-term effectiveness of hypopressive exercises on diastasis closure and LA improvement, in this case, we have chosen to start with rib opening and motor control to stimulate LA regeneration.

Some authors state that the DRA decreases spontaneously in the first 6-8 weeks postpartum [17], and in this case, the patient came for consultation at seven weeks postpartum, so the expected physiological improvements were already small without treatment.

After the conservative intervention, the patient continued to exercise at home while undergoing TRD. During the 10 TRD sessions, a decrease in the ARD of 1.9 cm at rest, 2.14 cm during supraumbilical contraction, and 1.78 cm during infraumbilical contraction was observed, reaching normal values at the end of treatment. Furthermore, the DI decreased from 69.29% to 18.48%. It can be assumed that the application of radiofrequency contributed to the improvement of the connective tissue in this case. The systematic review by González-Gutiérrez et al. [18] showed that TRD improves the strength of the pelvic floor muscles, which could also imply an improvement in the contractile strength of the rectus abdominis as well as the TrA and obliques. TRD is used for tissue repair in the range of 448-600 kHz. In this case, a frequency of 500 kHz was used as this is the ideal range to induce hyperthermia and stimulate the regeneration of muscle and tendon tissue. The LA is a tendinous and fibrous aponeurotic demarcation formed by the aponeurosis of the abdominal muscles. Capacitive/resistive TDR has been shown to be effective in the treatment of similar tissues with extracellular collagen matrix [19] due to its thermal effect. In response to this thermal increase, type I collagen fibrils are shortened. However, TRD therapy could currently deepen the tissue [20], which mainly provides resistance, and distribute its impedance between skin, connective tissue, and muscle. Given the separation of the rectus abdominis in DRA and the low abdominal adiposity in this patient, it is to be expected that TRD treatment may also have effects on the LA. Indeed, a study on athletes shows that TRD can improve the elasticity and contractile parameters of the knee flexors [21]. All these regenerative effects of LA tissue together with the prescribed therapeutic exercises could explain the reduction in DRA and DI.

## Conclusions

This case study demonstrated the potential efficacy of a therapeutic exercise in combination with TRD in reducing DRA and improving the patient's quality of life. These results suggest that TRD could be an effective complementary therapy for improving connective tissue integrity and LA functionality, especially in cases of severe diastasis following twin pregnancy.

However, as this is a single case study, the findings should be interpreted with caution. Further studies with larger sample sizes and more robust experimental designs are needed to confirm the efficacy of TRD as an adjunct treatment in the rehabilitation of DRA. The two different measurement systems can be a bias in the analysis of the results of the first part of the intervention. Furthermore, it would be necessary to conduct research only with TRD to confirm its efficacy.
